# Liver function in X-linked myotubular myopathy and autosomal dominant centronuclear myopathy: Data of the unite-CNM study

**DOI:** 10.1177/22143602251329215

**Published:** 2025-04-24

**Authors:** S Colombo, BS Cowling, L Eyler, D Nijkamp, C Freitag, L Thielemans, K Bouman, J Baets, J Vissing, R Quinlivan, M Guglieri, F Montagnese, U Schara-Schmidt, A Dhawan, MW Lawlor, NC Voermans

**Affiliations:** 1 Dynacure SA (now Flamingo Therapeutics NV), Louvain, Belgium; 2 Department of Neurology, Donders Institute for Brain, Cognition and Behavior, Radboud University Medical Center, Nijmegen, The Netherlands; 3 Department of Neurology, Neuromuscular Reference Centre, Antwerp University Hospital, Antwerp, Belgium; 4 Faculty of Medicine and Health Sciences, Translational Neurosciences, University of Antwerp, Antwerp, Belgium; 5 Laboratory of Neuromuscular Pathology, Institute Born-Bunge, University of Antwerp, Antwerp, Belgium; 6 Copenhagen Neuromuscular Center, Department of Neurology, Rigshospitalet, University of Copenhagen, Copenhagen, Denmark; 7 MRC Centre for Neuromuscular Diseases, UCL Institute of Neurology, London, UK; 8 John Walton Centre for Neuromuscular Disease, Newcastle University and Newcastle Hospitals NHS Foundation Trust, Newcastle-upon-Tyne, UK; 9 Friedrich Baur Institute at the Department of Neurology, LMU University Hospital, LMU Munich, Munich, Germany; 10 Department of Pediatric Neurology, Developmental Neurology and Social Pediatrics, University of Essen, Germany; 11 Pediatric Liver GI and Nutrition Centre and Mowat Labs, King's College Hospital, London, UK; 12 Diverge Translational Science Laboratory and Medical College of Wisconsin, Milwaukee, WI, USA

**Keywords:** liver involvement, steatosis, X-Linked myotubular myopathy and autosomal dominant centronuclear myopathy, peliosis

## Abstract

**Background:**

Centronuclear myopathies represent a subset of debilitating genetic disorders, for which no treatment exists. The Unite-CNM trial (NCT04033159) aimed to assess the effect of an antisense oligonucleotide to reduce *DNM2* mRNA expression in X-linked myotubular myopathy (XLMTM) and autosomal dominant centronuclear myopathy (ADCNM).

**Objective:**

The trial was discontinued due to tolerability concerns (hepatic and hematological). This report aims to provide an overview of hepatic involvement in XLMTM and ADCNM adults.

**Methods:**

The medical history and prospective liver imaging and liver function test results at screening and baseline were assessed. Furthermore, DNM2 protein expression in livers of four other pediatric patients with XLMTM and of healthy children and adults were assessed.

**Results:**

Twenty-six patients were screened; 15 with *DNM2* mutations (median age 36 years; six females), and 11 with *MTM1* mutations (median age 52 years; five females). Overall, six patients had a history of liver disease (6/19;31.6%). One patient with XLMTM had elevated serum alanine transaminase and another XLMTM patient had elevated serum gamma glutamyl transpeptidase. Liver ultrasound showed no features of peliosis hepatis. Liver steatosis was observed in three ADCNM patients and two XLMTM patients. The Fibroscan CAP score was above normal range in three XLMTM patients, and borderline or normal in other patients. The histopathology study showed that DNM2 protein levels in human liver decrease with age and are lower in pediatric individuals with XLMTM compared to controls.

**Conclusions:**

This study provides an overview of hepatic involvement in a large group of ADCNM and XLMTM patients. Findings suggest an underlying liver pathology may impact tolerability of therapeutic approaches, and will be important to consider for future trial design and clinical management. The results of DNM2 protein expression warrant further investigations on the role of DNM2 in the liver if it is to be used as a therapeutic target.

## Introduction

Centronuclear myopathies represent a subset of debilitating genetic disorders, that has been an active area of treatment development recently, particularly in the subsets of patients diagnosed with X-linked myotubular myopathy (XLMTM) or autosomal dominant centronuclear myopathy (ADCNM). XLMTM is a rare, life-threatening congenital myopathy caused by loss-of-function mutations in *MTM1*, located on the X chromosome, leading to absent or insufficient functional myotubularin, a protein required for normal development, maturation, and maintenance of muscle cells.^1^ XLMTM affects 1 in 32,000–40,000 newborn males,^2^ but accumulating evidence shows that carriers can display the disease with various degrees of severity.^3–5^ While XLMTM is most notably characterized by profound muscle weakness, resulting in severe respiratory distress at birth in the majority of patients, myotubularin is ubiquitously expressed and its functions outside of skeletal muscle are unclear. Similarly, ADCNM is a rare, predominantly early-onset, often congenital, myopathy resulting in gradually progressive difficulty with ambulation. Ptosis, ophthalmoplegia, facial weakness, dysphagia, and respiratory insufficiency are commonly reported, but generally manifest later in life.^6^ It is caused by mutations in *DNM2*, located on chromosome 19, suspected to lead to a gain of function of dynamin 2, a large ubiquitous GTPase protein with membrane fission and tubulation activities.^7^ The prevalence of ADCNM is estimated to be slight lower than that of XLMTM.^2^ Both MTM1 and DNM2 proteins act in a common pathway that regulates muscle function,^7^ however, their role in the liver is unknown. Liver dysfunction has not been studied systematically in XLMTM and ADCNM patients but has been a subject of several case studies.

Hepatic peliosis, a life-threatening vascular lesion characterized by blood-filled, and cystic cavities have been the primary liver abnormality reported in XLMTM, affecting 5–10% of patients.^8–13^ Due to the potential for hepatic peliosis to result in fatal or life-threatening hemorrhage, its presence affects the medical care of XLMTM patients in whom it is suspected. Therefore, efforts have been made to identify and exclude patients with hepatic peliosis in early treatment trials for XLMTM to prevent confounding safety signals from pre-existing lesions. Hepatic peliosis has not been reported in ADCNM, so this has not been an area of concern in ADCNM treatment studies. In addition to hepatic peliosis, XLMTM patients experience hepatobiliary disease more frequently than the general population, with a presentation of jaundice, cholelithiasis, pruritus, hepatomegaly, and elevated transaminases.^14^ Cholestatic disease in XLMTM has been under-recognized and under-studied, as it is usually not associated with significant morbidity or mortality in untreated XLMTM patients.^15^ The INCEPTUS Natural history study (a prospective, multicenter, non-interventional study in XLMTM on disease-related adverse events, respiratory and motor function, feeding, secretions, and quality of life (NCT02704273)) reported that 91% of participants (31 of 34 patients) showed some sign of hepatobiliary abnormality in their history or during the study.^16^ Next, in the ASPIRO trial (an open-label, dose-escalation trial with AT132, the investigational AAV8 gene replacement therapy for *MTM1* (NCT03199469; Astellas Gene Therapies, formerly Audentes Therapeutics), 24% of patients (8 of 34) had a history of hepatobiliary disease before or without treatment.^17,18^

In 2020 and 2021, four study participants with XLMTM treated with AT132 died following serious adverse events related to decompensated liver disease associated with cholestasis.^18^ The first three patients were among the eldest and heaviest participants in the study, receiving the highest total doses. The fourth patient received the lowest study dose, as the patient was younger, smaller, and received a lower dose as per body weight. These patients with fatal outcome experienced progressive cholestatic liver disease starting at three to four weeks following dosing, which led to death from complications of liver failure (either sepsis or hemorrhage) in the ensuing weeks to months.^19,20^ Given that these patients, and more than half of ASPIRO participants, had evidence of pre-existing hepatobiliary disease, and 62% of patients had adverse hepatic events, characterizing the underlying hepatic phenotypes related to myotubularin deficiency is highly pertinent. With respect to ADCNM, there have been no reports of cholestatic disease to date, however this likely requires further focused examination as the importance of cholestasis in XLMTM was under-recognized until recently.

These recent developments have led to the start of a number of (pre-)clinical studies on hepatic involvement in XLMTM and CNM as clinical trials in this area were forced to adapt to an unexpected class of safety findings.^15,21,22^ The urgency of understanding this hepatic involvement has become even greater since the Unite-CNM trial (NCT04033159; Dynacure), investigating an antisense oligonucleotide targeting *DNM2* for degradation, was terminated in 2022 due to tolerability issues, including liver enzyme abnormalities. Not only patients with XLMTM but also patients with ADCNM experienced transient an increase of transaminases and bilirubin in the weeks following treatment. Results of this trial have recently been published on EudraCT (https://www.clinicaltrialsregister.eu/ctr-search/trial/2018-004089-33/results) and Clinicaltrials.gov (https://clinicaltrials.gov/ct2/show/results/NCT04033159?term = dynacure&draw = 2&rank = 2).

In this paper, we aim to provide an overview of hepatic involvement in XLMTM and ADCNM adults in the Unite-CNM trial. We therefore report the data on the liver disease in the medical history, and prospective liver imaging and liver function test results at screening and baseline. Furthermore, we have investigated DNM2 protein expression in liver of healthy children and adults and in livers of four pediatric patients with XLMTM.

## Methods

### Unite-CNM study design

The present study is part of the study entitled ‘Phase 1/2 trial on the safety, tolerability, pharmacokinetics, pharmacodynamics and exploratory efficacy of DYN101 in patients ≥ 16 years of age with centronuclear myopathies caused by mutations in DNM2 or MTM1’ (https://www.clinicaltrialsregister.eu/ctr-search/trial/2018-004089-33/DK; https://clinicaltrials.gov/ct2/show/study/NCT04033159?term = dynacure&draw = 2&rank = 2). The investigational medicinal product (IMP) DYN101 was a generation 2.5 cEt gapmer antisense oligonucleotide targeting DNM2 for degradation. The study was performed in a total of 8 sites in 6 EU countries (Netherlands, Belgium, Germany, France, Denmark, UK). The first subject was enrolled on 04 March 2020. The last subject's visit was on 08 July 2022.

**Figure 1.**
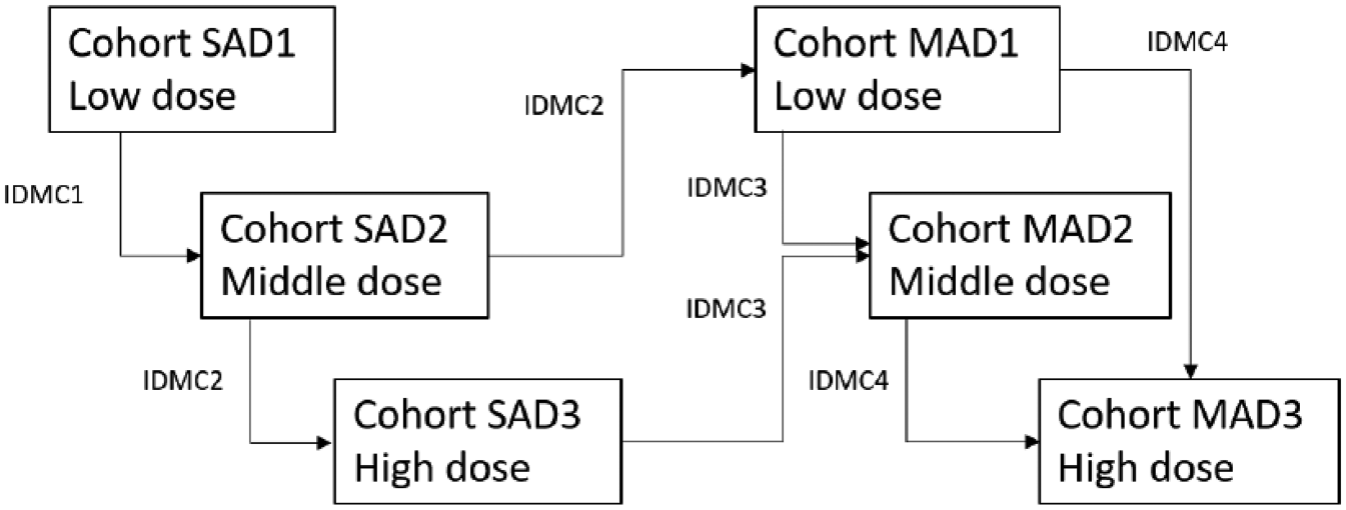
Unite-CNM study design algorithm.

Part 1 was a single-ascending dose (SAD) with a single dose of IMP at Day 1 with 4 weeks of follow-up after IMP administration and a washout period of at least 12 weeks. Part 2 was a multiple-ascending dose (MAD) of 12 weeks and a MAD extension part of 12 weeks with weekly doses of IMP. Subjects were to receive DYN101 at a low (1.5 mg/kg), middle (4.5 mg/kg), or high (up to 9 mg/kg) dose level in Cohorts 1, 2, and 3, respectively. All subjects were to participate in the SAD, MAD, and MAD extension parts, unless they withdrew (Figure 1). End of Treatment (EoT) assessments were to be performed after 24 weeks of MAD treatment had been completed, i.e., at the Week 25 visit. Subjects were to return to the clinic three months after the last IMP administration to follow-up on the subject's status including abnormal laboratory results, adverse events (AEs), and concomitant medications.

Part 1 was a first in human, single-ascending dose (SAD) with a single dose of IMP at Day 1 with 4 weeks of follow-up after IMP administration and a washout period of at least 12 weeks, in patients with MTM1 or DNM2 mutations. Part 2 was a multiple-ascending dose (MAD) of 12 weeks and a MAD extension part of 12 weeks with weekly doses of IMP. Subjects were to receive DYN101 at a low (1.5 mg/kg), middle (4.5 mg/kg), or high (up to 9 mg/kg) dose level in Cohorts 1, 2, and 3, respectively. Advancement to the next step was carried out only after approval from the independent data monitoring committee (IDMC). All subjects were to participate in the SAD, MAD, and MAD extension parts, unless they withdrew.

The protocol was amended repeatedly to be able to comply with the local regulations related to the COVID pandemic, and to ensure the safety of study participants after the data of hepatic adverse events in the Audentes trial had become publicly available, and liver and hematological safety data emerged in the course of the trial. These amendments included the introduction of liver ultrasound for ADCNM, liver elastography (Fibroscan) and more extensive and more frequent monitoring of liver and hematological parameters. The study was terminated during the MAD part of cohort 1.

### Liver function tests

The liver function tests (LFTs) were part of the laboratory safety assessments performed at screening, baseline and during the course of the trial. LFTs included LDH, AST, ALT, ALP, GGT, bilirubin (direct, indirect, and total), albumin and total serum bile acids (TBA). The schedule of safety assessments is included in the CSR. We here report the data of screening and baseline.

### Liver ultrasound and elastography

Liver imaging was part of the laboratory safety assessments performed at screening, and before starting the MAD part. A liver ultrasound and liver elastography (Fibroscan) to assess hepatic peliosis and other structural abnormalities were performed, initially in XLMTM patients but later in all participants. The results had to be available before administration of the IMP. Abnormalities identified at baseline were documented.

### Human liver samples

Eleven healthy liver autopsy samples (6 pediatric [newborn – 12 months] and 5 adult [60–79 years old]) were purchased from Tissue Solutions (BioIVT). Four pediatric XLMTM liver autopsy samples were obtained from the Congenital Muscle Disease Tissue Repository (CMD-TR) at Medical College of Wisconsin. Patients had given consent to histopathological analysis of these samples as part of the informed consent for trial participation.

### Western blot on human liver

Fifteen human autopsy liver tissues (6 healthy pediatric livers, 5 healthy adult livers, and 4 XLMTM pediatric livers) were available for protein analysis. Antibodies used were: primary mouse monoclonal antibodies against DNM2 (1/1000, #PA5-19800 – Thermo Scientific), or GAPDH (1/1000, #MA515738 – Invitrogen), goat anti-mouse secondary antibody coupled to horseradish peroxidase (1/1000 for GAPDH, #32430 – Invitrogen) and goat anti-rabbit secondary antibody coupled to horseradish peroxidase (1/1000 for DNM2, #32460 – Invitrogen). Details are available in the supplementary data.

### Statistical analysis

For the DNM2 protein expression, statistical analysis was performed using GraphPad Prism v9.4. The tests performed are indicated in the figure legends.

### Ethics statement

The studies were conducted in accordance with International Council for Harmonization (ICH) Good Clinical Practice (GCP) guidelines (CPMP/ICH/135/95) and the Declaration of Helsinki. Study protocols were approved by the regulatory agencies in each country, and subsequently institutional permission was granted by institutional review board / independent ethics committees (IRB/IECs). Signed informed consent forms (ICF) were obtained from participants or legal guardians. An independent data monitoring committee regularly monitored the integrity and safety of the trial. With respect to banked XLMTM tissue samples from non-trial patients that were obtained from the Congenital Muscle Disease Tissue Repository (CMD-TR), IRB oversight was provided by the Children's Hospital of Wisconsin (Project 407921-31).

## Results

### Study subjects

Patients were recruited for the Unite-CNM study by the PIs in the participating centres. Twenty-six patients were screened, 15 with *DNM2* mutations (ADCNM; 6 females) and 11 with *MTM1* mutations (XLMTM; 5 females). The mean age was 39.0 years (Table 1). We obtained the exact genotype of 22 of these individuals.

**Table 1.** Demographics of the 26 subjects screened for the unite-CNM trial, reason for screen failure and liver-related medical history.

Patient	Cohort	Mutation	Genotype	Age at time of IC	Sex	Ethnicity	Race	BMI (kg/m^2^)	Reason for screen failure	Screen failure liver related?	Liver history (start date)
1	1	DNM2	c.1393C > T (p.Arg465Trp)	34	F	Not Hispanic or Latino	White	18.7	N/A	N/A	None
2	1	DNM2	c.1393C > T (p.Arg465Trp)	51	M	Not Hispanic or Latino	White	27.9	N/A	N/A	Moderate Liver Steatosis (June 2018)
3	1	DNM2	c.1393C > T (p.Arg465Trp)	59	M	Not Hispanic or Latino	White	25.3	N/A	N/A	Liver Steatosis Grade 2 (July 2020)
4	1	MTM1	NR	52	F	Not Hispanic or Latino	NR	36	N/A	N/A	Hepatic Fibrosis (unknown) and Hepatic Steatosis (2016)
5	1	MTM1	c.1496G > T (p.Trp499Leu)	19	M	Not Hispanic or Latino	White	20.2	N/A	N/A	None
6	1	MTM1	c.695A > G (p.His232Arg)	24	M	Not Hispanic or Latino	White	17.4	N/A	N/A	Gilbert syndrome (November 2019)
7	2	DNM2	c.1393C > T (p.Arg465Trp)	36	M	Not Hispanic or Latino	White	24.5	N/A	N/A	None
8	2	MTM1	NR	53	F	Not Hispanic or Latino	NR	31.1	N/A	N/A	None
9	2	DNM2	c.1105C > T (p.Arg369Trp)	41	M	Not Hispanic or Latino	White	26.6	N/A	N/A	None
10	2	DNM2	c.1106G > A (p.Arg369Gln)	54	M	Not Hispanic or Latino	White	23.4	N/A	N/A	None
11	2	MTM1	c.1210G > A (p.Glu404Lys)	20	M	Not Hispanic or Latino	White	19.9	N/A	N/A	Gilbert syndrome (unknown)
12	2	MTM1	c.1354-2A > T (r.spl?)	58	F	Not Hispanic or Latino	White	26.4	N/A	N/A	None
13	2	DNM2	c.1393C > T (p.Arg465Trp)	19	F	Not Hispanic or Latino	White	25.8	N/A	N/A	None
14	2	DNM2	c.1105C > T (p.Arg369Trp)	18	F	Not Hispanic or Latino	White	22.1	N/A	N/A	None
15	Screen failure	DNM2	c.1565G > A (p.Arg522His)	64	F	Not Hispanic or Latino	White	21.2	Study Terminated by Sponsor	N/A	NR
16	Screen failure	MTM1	NR	25	M	Not Hispanic or Latino	NR	24.3	Failure to Meet Enrollment Criteria	Yes	NR
17	Screen failure	MTM1	NR	57	F	Not Hispanic or Latino	NR	NR	Failure to Meet Enrollment Criteria	No	NR
18	Screen failure	DNM2	NR	26	M	Not Hispanic or Latino	NR	NR	Withdrawal by Subject	N/A	NR
19	Screen failure	MTM1	c.1210G > A (p.Glu404Lys)	18	M	Not Hispanic or Latino	White	NR	Failure to Meet Enrollment Criteria	Yes	Cholinergic crisis (2019) unknown if intra- or extra-hepatic
20	Screen failure	DNM2	c.1106G > A (p.Arg369Gln)	19	M	Not Hispanic or Latino	White	28	Physician Decision	Yes	None
21	Screen failure	DNM2	c.1393C > T (p.Arg465Trp)	36	M	Not Hispanic or Latino	White	NR	Failure to Meet Enrollment Criteria	Yes	None
22	Screen failure	MTM1	ChrX(GRCh37)g.149680273_ 150154664del; heterozygous (Deletion removes three genes: MTM1, MTMR1, CD99L2)	61	F	Not Hispanic or Latino	White	NR	Failure to Meet Enrollment Criteria	Yes	NR
23	Screen failure	MTM1	c.208C > T (p.Leu70Phe)	58	M	Not Hispanic or Latino	White	NR	Failure to Meet Enrollment Criteria	No	Fatty liver disease (2005)
24	Screen failure	DNM2	c.1565G > A (p.Arg522His)	38	F	Not Hispanic or Latino	White	NR	Failure to Meet Enrollment Criteria	No	None
25	Screen failure	DNM2	c.1102G > A (p.Glu368Lys)	20	F	Not Hispanic or Latino	White	14.8	Study Terminated by Sponsor	N/A	NR
26	Screen failure	DNM2	c.1565G > A (p.Arg522His)	53	M	Not Hispanic or Latino	White	NR	Withdrawal by Subject	N/A	NR

A total of 14 subjects were randomized: 6 subjects in Cohort 1 (three subjects with a mutation in DNM2 and three subjects with a mutation in MTM1) and 8 subjects in Cohort 2 (five subjects with a mutation in DNM2 and three subjects with a mutation in MTM1). Twelve subjects were screen failures.

IC: Informed Consent; NR: not reported; N/A: not applicable. Blue: underweight; Orange: overweight; Red: obese.

Among the 14 ADCNM patients, the missense mutations included p.Arg465Trp (n = 6), p.Arg522His (n = 3), p.Arg 369Trp (n = 2) and p.Arg369Gln (n = 2), and p.Glu368Lys (n = 1) (Table 1). Among the 7 XLMTM patients, the missense mutations in *MTM1* included p.Glu404Lys (n = 2), p.Trp499Leu (n = 1), p.His232Arg (n = 1) and p.Leu70Phe (n = 1). One patient carried a splice-site mutation and another one carried a deletion removing the genes *MTM1*, *MTMR1* and *CD99L2* (Table 1).

The median age was 36 years for ADCNM patients and 52 years for XLMTM patients (Table 2). There were nine males and six females in the ADCNM subcohort, and six males and five females in the XLMTM subcohort, confirming the potentially higher number than previously thought of symptomatic carriers with XLMTM (Table 2). All patients were reported as white (Table 2). The median BMI was 24.5 kg/cm^2^ and 24.3 kg/cm^2^ for ADCNM and XLMTM patients, respectively (Table 2). One ADCNM (1/11 [9%]) and one XLMTM (1/7 [14.3%]) patient were underweight, while four ADCNM (4/11 [36.4%]) and two XLMTM (2/7 [28.6%]) patients were overweight, and two XLMTM (2/7 [28.6%]) patients were obese (Table 1).

**Table 2.** Summary demographics and clinical characteristics of the subjects screened for the unite-CNM trial.

Parameter	ADCNM (*DNM2* mutations)	XLMTM (*MTM1* mutations)
Age in years	36 [18–64]; n = 15	52 [18–61]; n = 11
Gender (Male:Female)	9:6; n = 15	6:5; n = 11
Race	14 white, 1 not reported	8 white, 3 not reported
Body mass index in kg/m^2^	24.5 [14.8–28]; n = 11	24.3 [17.4–36]; n = 7
Liver-related medical history	2; n = 11 (18%)	4; n = 8 (50%)
Screen failure liver-related	2; n = 3 (67%)	3; n = 5 (60%)

Values are expressed as median [range] for Age and Body Mass Index; n = number of patients analyzed.

Of the 26 screened patients, a total of 14 subjects were enrolled and randomized as follows: six subjects in Cohort 1 (three subjects with a mutation in *DNM2* and three subjects with a mutation in *MTM1*) received a single intravenous injection of DYN101 at the low 1.5 mg/kg dose during the SAD1part, and weekly injections of DYN101 at the 1.5 mg/kg dose during the MAD1 part; 8 subjects in Cohort 2 (five subjects with a mutation in *DNM2* and three subjects with a mutation in *MTM1*) received a single intravenous injection of DYN101 at the middle 4.5 mg/kg dose during the SAD2 part. The trial was terminated before these subjects started the MAD2 part. Cohort 3 at the high dose had not started before the study was terminated.

None of the subjects completed the study. “Study terminated by sponsor” was the most common reason for non-completion (6 [100.0%] subjects in Cohort 1 and 7 [87.5%] subjects in Cohort 2, respectively). One subject in Cohort 2 terminated early following physician decision (Table 1).

Twelve subjects were labelled as screen failures (seven with ADCNM and five with XLMTM). Of these, two subjects were not enrolled due to study terminated by sponsor (16.7%), two subjects withdrew from the study (16.7%), seven subjects failed to meet enrollment criteria (58.3%), and one subject was not enrolled due to physician decision (8.3%). Four out of the seven subjects who failed to meet enrollment criteria and the subject who was not enrolled due to physician decision were labelled as screen failures due to liver-related issues (five out of eight subjects [62.5%]; two with ADCNM and three with XLMTM) (Tables 1 and 2).

### History of liver disease

We obtained liver-related medical history for the 14 patients that were enrolled in Cohorts 1 and 2 as well as 5 screen failures (Tables 1 and 2). Overall, six patients out of 19 screened presented with a history of liver disease (31.6%). Two subjects with ADCNM had moderate hepatic steatosis (2/11 [18%]), one subject with XLMTM had hepatic fibrosis as well as hepatic steatosis (1/8 [12.5%]), another subject with XLMTM had hepatic steatosis (1/8 [12.5%]), and two subjects with XLMTM had Gilbert syndrome (2/8 [25%]), a common benign condition where the liver does not conjugate bilirubin. Nine subjects with ADCNM and four subjects with XLMTM did not have any known liver disease.

### Liver function tests

The results of the liver function tests during screening, at SAD baseline and at MAD baseline, are reported in (Table 3). All patients had normal serum alanine transaminase (ALT), except one with XLMTM (patient #16) with values 1.6x the upper limit of normal (ULN) at 75 IU/L and 73 U/L during two screening visits. All patients had normal aspartate transaminase (AST). Lactate Dehydrogenase (LDH) was also normal in the 14 patients that were tested (10 with ADCNM and four with XLMTM). One patient with XLMTM (patient #22) had serum Gamma glutamyl transpeptidase (GGT) values 1.5x ULN (103 IU/L and 93 IU/L at screening and Run-In Visit, respectively). This patient had normal bilirubin levels. Serum alkaline phosphatase (ALP) levels showed a mild elevation in 8 subjects, consisting of values below 150, which were as not to be of pathological significance given normal GGT values. Two patients with XLMTM (patients #6 and #11) had serum total bilirubin at or above the ULN (21 µmol/L and 27 µmol/L at screening, respectively) due to genetically confirmed Gilbert syndrome. The conjugated fraction of these total bilirubin values was not tested during screening and SAD baseline but both patients had normal serum GGT values. For patient #6, conjugated and unconjugated bilirubin were tested at MAD baseline and were also above the ULN (conjugated: 9 µmol/L; unconjugated: 15 µmol/L). The serum total bile acid values were normal in 10 patients with ADCNM and 4 patients with XLMTM in whom it was measured. Albumin levels were also normal in all patients.

**Table 3.** Results of liver function tests at screening and baseline.

Patient	Cohort	Sex	Mutation	Visit	Date	ALT (U/L)	AST (U/L)	LDH (U/L)	ALP (U/L)	GGT (U/L)	Bilirubin direct (µmol/L)	Bilirubin indirect (µmol/L)	Bilirubin total (µmol/L)	Total bile acids (µmol/L)	Albumin (g/L)
1	1	F	DNM2	Screening	27-Feb-20	9	13		52	10			5		49
				SAD baseline	3-Mar-20	9	12		50	10			4		46
				Unscheduled	10-Dec-21	10	13	115	57	14	3	4	7		49
				MAD baseline	27-Dec-21	6	12	114	53	12	3	2	5	4	49
2	1	M	DNM2	Screening	9-Mar-20	33	18		23	29					44
				Scr Unscheduled	23-Jun-20	26	18		24	23			5		43
				SAD baseline	7-Jul-20	30	19		22	24			4		42
				Unscheduled 1	21-Dec-21	32	21						3	2.3	42
				Unscheduled 2	21-Dec-21	37	19	127	32	25	2	3	5		46
				MAD baseline	17-Jan-22	30	17	127	30	25	3	3	6	3	42
3	1	M	DNM2	Screening	22-Sep-20	20	18		65	19			8		39
				SAD baseline	13-Oct-20	19	18						9		44
				Unscheduled	11-Jan-22	29	26		62	14	3	5	8		44
				MAD baseline	31-Jan-22	28	23	134	56	14	5	5	10	1.6	42
4	1	F	MTM1	Screening	29-Jul-20	23	22		93	17			8		43
				Scr Unscheduled 1	17-Aug-20	15	14		91	17			9		39
				Scr Unscheduled 2	5-Oct-20	19	19		87	15			13		40
				SAD baseline	3-Nov-20	17	15		90	15			6		38
				Unscheduled	15-Dec-21	22	19	216	104	16	4	6	10	1.6	44
				MAD baseline	24-Jan-22	20	17	205	102	17	3	5	8	1.7	41
5	1	M	MTM1	Screening	18-Feb-20	18	21		68	26			10		43
				Scr Unscheduled 1	25-Aug-20	15	20		73	32			8		46
				SAD baseline	31-Aug-20	20	22		80	38			9		49
				Unscheduled 1	23-Nov-21	22	22	156	72	33	3	5	8	3.7	48
				Unscheduled 2	18-Jan-22	18	21	162	70	38	3	4	7	5.1	48
				MAD baseline	31-Jan-22	16	21	163	69	33	4	4	8	1.8	46
6	1	M	MTM1	Screening	6-Nov-20	27	18		73	23			21		46
				SAD baseline	23-Nov-20	24	18		74	21			21		45
				Unscheduled	26-Jan-22	39	25		90	24	9	22	31	4.1	47
				MAD baseline	14-Feb-22	26	18	122	90	25	9	15	24		51
7	2	M	DNM2	Screening	1-Dec-20	23	18		47	10			11		45
				Run-in Visit 1	29-Dec-20	19	14		46	9			12		42
				SAD baseline	15-Feb-21	22	21		51	9			7		45
8	2	F	MTM1	Screening	20-Sep-21	24	21		55	23			5		38
				Scr Unscheduled	7-Dec-21	29	24	208	61	23	2	1	3	3.6	42
				SAD baseline	3-Jan-22	24	20	196	54	21	2	2	4	2.4	39
9	2	M	DNM2	Screening	16-Sep-20	29	15		70	41			6		46
				Run-in Visit 1	15-Oct-20	27	17						4		46
				Run-in Visit 2	14-Jan-21		16						4		44
				SAD baseline	8-Mar-21	23	15		67	36			6		44
10	2	M	DNM2	Screening	19-Jan-21	19	14		61	12			8		45
				Run-in Visit 1	1-Mar-21	19	14		58	10			4		42
				Run-in Visit 2	31-May-21	17	14		63	12			5		42
				SAD baseline	17-Aug-21	29	19		58	13			9		40
11	2	M	MTM1	Screening	30-Sep-20	16	15		58	24			27		46
				Run-in Visit 1	3-Nov-20	18	15		59	24			27		48
				Run-in Visit 2	19-Jan-21	19	16		56	22			23		48
				SAD baseline	1-Feb-21	16	16		61	22			27		46
12	2	F	MTM1	Screening	20-Apr-21	20	21		96	17			7		41
				Run-in Visit 1	18-May-21	23	22		99	17			5		44
				SAD baseline	28-Jun-21	26	25		107	22			8		46
13	2	F	DNM2	Screening	18-Feb-20	20	19		63	9			12		45
				Scr Unscheduled	11-Jan-21	14	16		55	8			11		43
				SAD baseline	8-Feb-21	14	17		65	10			11		45
14	2	F	DNM2	Screening	30-Sep-20	11	18		53	10			4		46
				Run-in Visit 1	3-Nov-20	12	18		56	8			7		44
				Run-in Visit 2	25-Jan-21	10	16		56	8			6		45
				Run-in Visit 3	13-Apr-21	10	13		40	10			4		41
				Run-in Visit 4	5-Jul-21	10	14		39	10			6		43
				Run-in Visit 5	25-Oct-21	10	16		43	10			0		44
				SAD baseline	29-Nov-21	9	16	132	40	9	4	4	8	4.3	44
15	Screen failure	F	DNM2	Screening	7-Feb-22	25	20	148	113	7	2	2	4	8.3	40
				Run-in Visit 1	7-Mar-22	26	21	152	100	7	1	0	0		40
16	Screen failure	M	MTM1	Screening	5-Oct-20	75	31		87	47			5		45
				Scr Unscheduled	21-Oct-20	73	30		90	36			7		45
17		F	MTM1	Screening	21-Apr-21	24	17		55	25			6		39
18	Screen failure	M	DNM2	Screening	14-Mar-22	23	24		65	8			11		45
				Scr Unscheduled	22-Mar-22	17	13	128	63	10	3	4	7	4.2	45
19	Screen failure	M	MTM1	Screening	17-Feb-22	23	22		112	7			5		46
20	Screen failure	M	DNM2	Screening	7-Jul-21	19	15		146	9			7		46
				Run-in Visit 1	13-Aug-21	21	17		138	9			9		41
				Run-in Visit 2	19-Nov-21	20	15	145	144	10	4	6	10	0	44
21	Screen failure	M	DNM2	Screening	1-Sep-21	39	24		61	17			8		44
22	Screen failure	F	MTM1	Screening	23-Feb-21	35	12		114	103			4		41
				Run-in Visit 1	23-Mar-21	38	14		96	93			4		41
23	Screen failure	M	MTM1	Screening	24-Jun-21	35	30		80	24			6		43
				Scr Unscheduled	14-Dec-21	22	19		105	24			5		38
24	Screen failure	F	DNM2	Screening	23-Mar-22	7	12	129	70	9	7	13	20	7.4	45
25	Screen failure	F	DNM2	Screening	24-Jan-22	15	15	127	70	6	2	3	5	5.4	48
				Run-in Visit 1	10-Mar-22	20	16	122	60	5	3	2	5	4.7	45
26	Screen failure	M	DNM2	Screening	15-Mar-22	25	19	130	96	12	3	3	6	8.8	39

Reference values [LLN-ULN]: ALT [0–45]; AST [0–41]; LDH [100–242] for males and [100–220] for females; ALP [40–129] for males and [35–104] for females; GGT [2–65]; bilirubin direct [0–7]; bilirubin indirect [0–12]; bilirubin total [0–21]; total bile acids [0–10]; albumin [32–55]. Scr: Screening; ULN: Upper Limit of Normal; LLN: Lower Limit of Normal; Red: value above ULN; Orange: value = ULN; Blue: below LLN.

### Liver ultrasound examination

**Figure 2.**
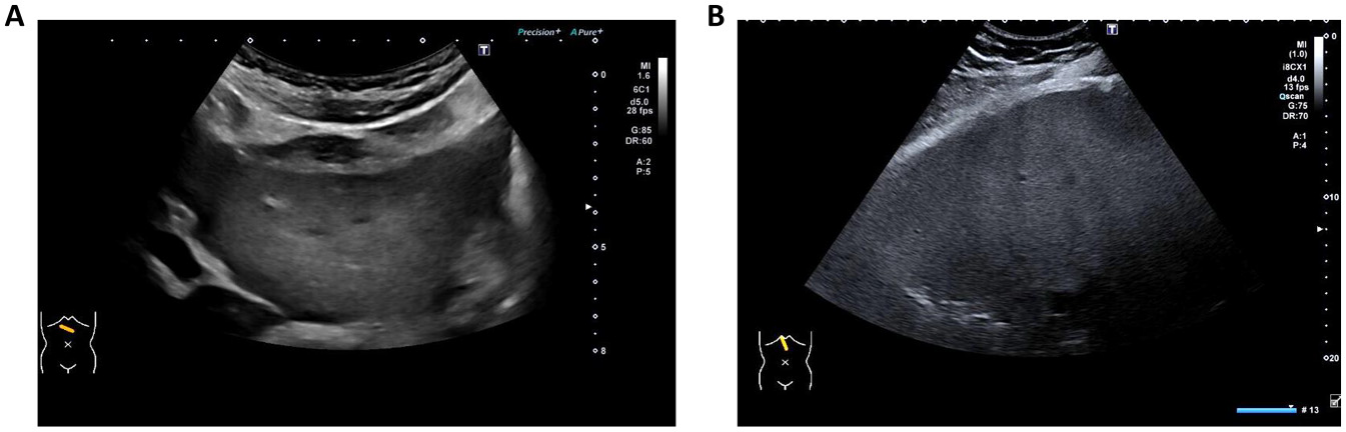
Liver ultrasound.

On ultrasound assessment of the liver, none of the patients had features of peliosis hepatis. Ultrasound features suggesting a fatty infiltration were seen in three patients with ADCNM (patients #2, #21 and #24) and two patients with XLMTM (patients #22 and #23). None of these patients had an elevation in liver transaminases. Corresponding controlled attenuation parameter (CAP), which reflects the level of fatty change in the liver, was not available in three of these patients and borderline to normal in the other two. None of the patients showed abnormal liver architecture or mass lesion on ultrasound. Two patients had a mild splenomegaly on ultrasound (patients #21 and #22; Figure 2, Table 4).

(A) Normal liver ultrasound of 20-year-old male subject with XLMTM (patient #11). (B) Mild steatosis on liver ultrasound of 61-year-old female subject with XLMTM: mild increase of echo intensity and lightly impaired appearance of the portal vein wall and the diaphragm (patient #22).

**Table 4.** Summary of liver imaging findings.

			Ultrasound	Fibroscan		
Patient	Cohort	Mutation	Summary	LSM (median)	CAP (median)	Summary
1	1	DNM2	Normal	2.1 kPa; IQR/Med: 38%	Not assessed / not reported	F-score 0; S-grade 1
2	1	DNM2	Moderate liver steatosis (grade 2): increase in echogenicity of the liver, loss of echogenicity at the border of portal veins	4.4 kPa; IQR: 0.6; IQR/Med: 14%	235 dB/m; IQR 12	F-score 0; S-grade 1
3	1	DNM2	Normal	8.37 kPa; IQR: 5; IQR/Med: 60.2%	Not assessed / not reported	No conclusion; technical issues
4	1	MTM1	Abnormal, not clinically significant*	6 kPa*	323 dB/m*	F-score 1; S-grade 3. Abnormal, not clinically significant, minimal fibrosis*
5	1	MTM1	Normal, moderately echogenic liver	5.5 kPa; IQR: 1.5; IQR/Med: 28%	Not assessed / not reported	F-score 0-1; S-grade 1
6	1	MTM1	Normal	1st scan: 10.1 kPa 2nd scan: 6.7 kPa; IQR:- < 0.3; IQR/Med: < 4.5%	Not assessed / not reported	1st scan: Measurements are in keeping with cirrhosis (F-score 4), however this represents a discrepancy with the normal sonographic liver appearances. 2nd scan (2 weeks later): Significant fibrosis (F-score 2); S-grade not reported
7	2	DNM2	Not performed			Not performed
8	2	MTM1	Normal*	6.6 kPa*	237 dB/m*	F-Score 1; S-grade 1. Abnormal, not clinically significant, minimal fibrosis*
9	2	DNM2	Not performed			Not performed
10	2	DNM2	Normal, mild splenomegaly	4.73 kPa	Not assessed / not reported	F-score 0; S-grade 1
11	2	MTM1	Normal			Not performed
12	2	MTM1	Normal as far as can be assessed (Ultrasound very moderately accessible due to examination in a lying position in a wheelchair. Deliver intercostal approach only)	Median: 6.5 kPa; IQR: 1.2; IQR/Med: 18%	276 dB/m; IQR 17	F-score 0-1; S-grade 2; mild fibrosis and steatosis
13	2	DNM2	Not performed			Not performed
14	2	DNM2	Normal	3.3 kPa; IQR: 0.3; IQR/Med: 9%	148 dB/m; IQR 26	F-score 0-1; S-grade 1
15	Screen failure	DNM2	Normal, mild splenomegaly	Not assessed / not reported	Not assessed / not reported	Not measurable. Difficulty scanning due to “elevated liver”
16	Screen failure	MTM1	Normal*			Not performed / not reported*
17	Screen failure	MTM1	Not performed / not reported*			Not performed / not reported*
18	Screen failure	DNM2	Not performed / not reported*			Not performed / not reported*
19	Screen failure	MTM1	Performed but not reported	5.6 kPa; IQR: 1.6; IQR/Med: 29%	298 dB/m; IQR 45	F-score 1; S-grade 2-3. Indication of steatosis
20	Screen failure	DNM2	Mildly elevated echogenicity	9.8 kPa; range 9.0–12.5 kPa Repeat scan: 5.2 kPa	76 dB/m; range 67–80 dB/m Repeat: 332 dB/m	F-score 1-2; S-grade 3. Steatosis
21	Screen failure	DNM2	Steatosis: enlarged liver with elevated echogenicity. Fibrosis. Splenomegaly	11.8 kPa; range 10.9–15.1 kPa	87 dB/m; range 85–90 dB/m	F-score 2; S-grade 1
22	Screen failure	MTM1	Steatosis: Moderately echogenic liver. Splenomegaly	Not measurable	Not measurable	Not measurable. Fibroscan failed due to patient's habitus. Only M-probe present.
23	Screen failure	MTM1	Diffuse steatosis: Increased echogenicity, and slightly irregular contour (Limited liver views due to overlying bowel gas and patient's body habitus).	7 kPa	Not assessed / not reported	F-score 2; S-grade not reported. Portal fibrosis with a few septa
24	Screen failure	DNM2	Steatosis: Slight increase in echogenicity	6.6 kPa; IQR/Med: 21.2%	Not assessed / not reported	F-score 2; S-grade not reported. Portal fibrosis
25	Screen failure	DNM2	Normal			Not performed
26	Screen failure	DNM2	Normal			Not performed

The protocol changed throughout the study due to which the US / Fibro scan was made at different time points for different participants. It is therefore not correlated to liver function tests.

* Info from CRF. Site did not respond to request for information.

Red: above upper limit of normal; Orange: borderline values

LSM: Liver stiffness measurement; CAP: Controlled Attenuation Parameter; IQR: Interquartile Range of the median; IQR/Med: Interquartile range of the median expressed as a ratio or %.

### Liver elastography (fibroscan)

In patients in whom the CAP score was available, it was above normal range at 323 dB/m (patient #4), 276 dB/m (patient #12), and 298 dB/m (patient #19) in three patients with XLMTM, and within normal range, although borderline, at 237 dB/m (patient #8) for one patient with XLMTM. It was within normal range, although borderline, at 235 dB/m (patient #2), and normal at 148 dB/m (patient #14), 87 dB/m (patient #21) and 76 dB/m (patient #20) in four patients with ADCNM. However, for patient #20, a repeat scan showed an elevated CAP score of 332 dB/m, and the physicians reported liver steatosis as the cause of screen failure (Table 4).

The liver stiffness measurement (LSM) reflects the level of fibrosis in the liver. Valid LSMs with interquartile range of the measurements <30% were available in nine patients. In five patients with ADCNM, the median LSM was 6.6 kPa (range 3.3–11.8 kPa) while the median LSM in four patients with XLMTM was 6.05 kPa (range 5.5–6.7 kPa). Two patients with ADCNM (patient #20 and #21) had significant fibrosis LSM >7 kPa (9.8 kPa and 11.8 kPa, respectively). Patient #21 also had steatosis on ultrasound (Table 4).

### Liver-related TEAEs during the unite-CNM trial

Abnormal liver function tests were the most common treatment-emergent adverse events (TEAEs). Importantly no TEAEs resulted in clinical symptoms of liver dysfunction, and all values returned to normal after stopping the treatment. However, DYN101 was not well tolerated enough to continue dosing to reach a level required to achieve potential long term therapeutic benefit in patients, and thus development was stopped.

(See CSR on EudraCT (https://www.clinicaltrialsregister.eu/ctr-search/trial/2018-004089-33/results) and Clinicaltrials.gov (https://clinicaltrials.gov/study/NCT04033159?term = NCT04033159&rank = 1&tab = results#publications).

Direct and indirect bilirubin, total serum bile acids and LDH measurements were added to the protocol from November 2021. Thus, there was no testing for these parameters for the six cohort 1 patients at screening and SAD1 baseline, nor for six cohort 2 patients at screening and SAD2 baseline. All six cohort 1 patients were tested at MAD1 baseline, and two cohort 2 patients were tested at SAD2 baseline. Additionally, more frequent testing was introduced at the same time for all liver parameters: before the amendment, testing occurred at baseline, and Days 15, 29, 57, 85 and 113 for the SAD part; after the amendment, testing at Days 1, 2, 4, 8, and 11 were added. The same time points were added for the MAD1 part, with weekly testing occurring from Day 15.

### DNM2 expression in liver

**Figure 3.**
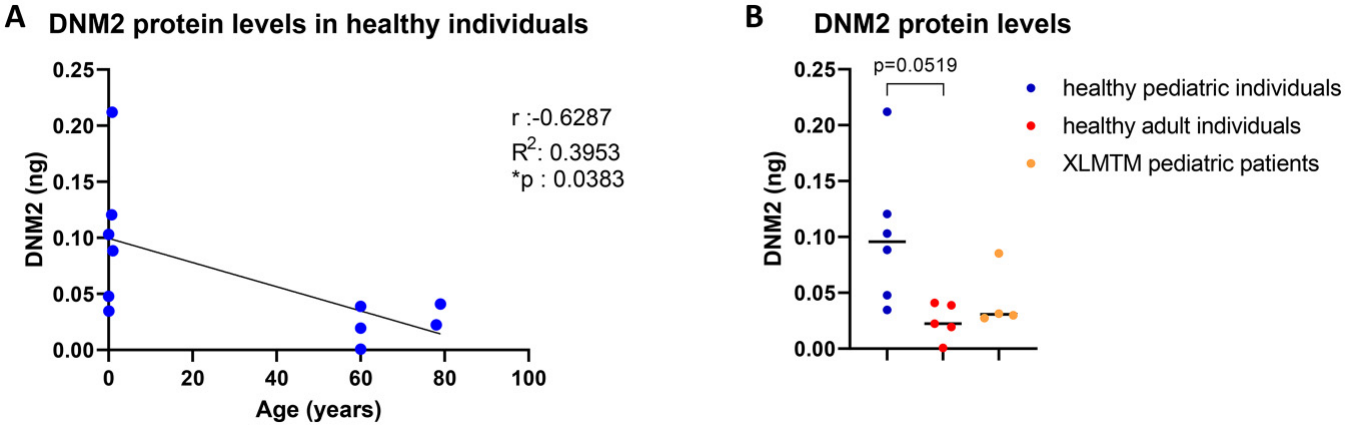
DNM2 protein levels in liver.

Since the IMP, DYN101, aimed to reduce *DNM2* expression, we decided to look at baseline DNM2 protein expression in human livers, in both adult and pediatric healthy livers and pediatric XLMTM livers, as this had not been studied previously. We observed that in the healthy population, DNM2 protein levels in the liver decrease with age (Figure 3(A)), as was previously observed in skeletal muscle^23^ (*personal communication, Dynacure*). In XLMTM pediatric patients, unlike in skeletal muscle where DNM2 protein levels are elevated,^24^ we observed that levels of DNM2 protein were not elevated compared to pediatric healthy individuals, with a mean expression similar to the adult healthy population (Figure 3(B)).

Western Blot analysis of DNM2 protein levels in healthy and CNM livers. (A) DNM2 protein levels in healthy livers from autopsy from pediatric and adult individuals, by age. A Pearson correlation test was performed. (B) DNM2 protein levels in healthy livers from autopsy from pediatric and adult individuals, and in livers from autopsy from XLMTM pediatric patients. An ordinary one-way ANOVA on log-transformed data was performed, followed by a Sidak's multiple comparisons test.

## Discussion

This data presents an overview of the results of medical history, liver function tests, liver imaging and elastography in adult XLMTM and ADCNM patients. These measurements were part of the baseline visits of the Unite-CNM study that had to be terminated because of tolerability issues. We thus provide an overview of hepatic involvement in a large group of adult CNM patients. Furthermore, we have investigated DNM2 expression in livers of four pediatric patients with XLMTM and of healthy children and adults. We discuss the main findings below.

Evidence is emerging, that a proportion of XLMTM patients experience liver symptoms at some point, including hepatomegaly, raised transaminases, alkaline phosphatase and GGT, cholestatic jaundice with or without pruritis, cholelithiasis, acute cholecystitis, hepatic steatosis and peliosis hepatis. Table 5 summarizes the key findings of these studies in XLMTM and ADCNM.

**Table 5.** Key findings of previous studies on liver involvement in CNM.

Authors & Study (year)	Population (n)	Key findings
Amburgey et al. Neurology 2017^25^	XLMTM (n = 50), 0–42 years	Hepatic manifestations in XLMTM with abnormal serum liver enzymes in 22.5%, hepatomegaly in 11.8%, jaundice in 14.7% and liver hemorrhage in 5.9% Hepatic peliosis can lead to death due to bleeding in the liver, which may occur spontaneously or be triggered, for example by liver biopsy.
D’Amico et al. Orphanet J Rare Dis 2021^21^	XLMTM (n = 12), 0–18 years	Elevated serum transaminases with gallstones and abnormal liver structure (including abnormal echogenicity or hemorrhagic spaces) on imaging in 42% and 58% of patients, respectively. Abnormalities were non-progressive without correlation to age, disease duration, clinical severity or type of MTM1 mutation.
Dowling et al. J Neuromuscul Dis 2022^16^ INCEPTUS	XLMTM (n = 34), < 4 year, ventilator support	91% Of patients had a history of hepatic disease and/or prospectively experienced related adverse events, or laboratory or imaging abnormalities across all three mutation types (nonsense, missense or in-frame exomic deletion) Patients with nonsense mutations had the most prevalent and pronounced abnormalities
Hayes et al. Neurol Genet 2022^6^	ADCNM (n = 42), 0–75 years	Liver function tests were abnormal in 4 patients, with ALT between 1 and 1.5 ULN in patients and AST between 1 and 1.2 ULN in two patients
Molera et al. J Neuromuscul Dis 2022^15^	XLMTM (n = 5), 5–16 months	Episodes of intrahepatic cholestasis in their disease natural history. following intercurrent infections or routine childhood vaccination These episodes presented with pruritus, hypertransaminemia, and hyperbilirubinemia with normal gamma-glutamyl transferase. The disease course was similar to benign recurrent hepatic cholestasis Genetic mutations associated with familial cholestasis were excluded (n = 3) Progression to cirrhotic, decompensated liver disease occurred (n = 1)
Fetalvero KM Mol Cell Biol 2013^26^	Mtm1-deficient mice	Liver manifestations may be related to defective autophagy and impaired lipid layer trafficking

The available data from the patients screened in the Unite-CNM trial show minimal or no hepatic involvement in most patients. The assessments showed evidence of liver injury in the form of an elevation of GGT and steatosis in one MTM1 patient, and elevated LSM with steatosis in two DNM2 patients. Three other patients had isolated steatosis on ultrasound, one MTM1 and two DNM2. Within the constraints of the available information, at least two of them may be assumed to have some form of cholestatic injury. The LSMs of 11.8 kPa (patient 21) and 9.8 (patient 20) indicate significant fibrosis along with steatosis. It is not known if the steatosis in this patient is a part of steatotic liver disease or of a metabolic derangement in DMN2-CNM, as data on Body Mass Index, insulin resistance or lipid profiles are unavailable to exclude unrelated metabolic associated steatotic liver disease. The lower frequency of hepatic derangement in the patients screened in this trial compared to the ASPIRO study is noteworthy. This may be related to the older age group in this cohort of patients with possibly milder phenotype of muscle disease and the fact that almost half of the patients with XLMTM were carriers. Existing reports do not support the conclusion that the frequency of liver manifestations has any correlation to the severity of the muscular disorder or patients’ age in this adult cohort.

Two patients with XLMTM had serum total bilirubin at or above the ULN due to Gilbert syndrome. This is a relatively common, harmless liver condition in which the liver does not conjugate bilirubin. It is caused by homozygous or compound heterozygous mutations in the UDP-glucuronosyltransferase gene (*UGT1A1*). An estimated 3% to 7% of the population have Gilbert syndrome. This abnormality is more common in males than in females. It affects all ages, races and ethnicities usually symptomatic with jaundice and fatigue in teenage years, no specific treatment is needed as the condition is considered of no pathological significance. Often, it is discovered incidentally when a blood test shows raised unconjugated bilirubin levels, as was the case in this trial.

The protocol of Unite-CNM was amended repeatedly to ensure the safety of study participants after the data of hepatic side effects in the ASPIRO trial had become publicly available, and liver and hematological safety data emerged in the course of the trial. This is likely to have contributed to the early detection of hepatic lab value abnormalities, the timely termination and complete recovery of lab values to normal range in study participants.

The results of the analyses of liver tissues in the healthy population showed a decrease of DNM2 protein levels with age. In liver tissues of pediatric XLMTM patients, levels of DNM2 protein tended to be lower than in pediatric healthy individuals. Unfortunately, we did not have access to adult XLMTM liver samples, nor liver samples from patients with ADCNM. Nevertheless, the results in pediatric biopsies suggest that DNM2 levels may be low at baseline in the XLMTM and ADCNM populations, however further investigation analyzing DNM2 levels in the livers of additional XLMTM and CNM patients across various ages is warranted.

In skeletal muscle, preclinical evidence suggests that MTM1 and DNM2 function in the same cellular pathway, and that elevated DNM2 expression and/or activation in skeletal muscle contributes to disease pathology in this tissue in various forms of centronuclear myopathies.^7,24^ In contract limited research is available on the role of MTM1 and DNM2 in liver (see below) and warrants further research, to better understand the impact of DNM2 reduction in the liver of XLMTM patients.

Recently, it was shown that loss-of-function of *MTM1* in zebrafish leads to cholestatic disease in these animals, and that a DNM2 inhibitor, Dynasore, was able to partially restore the disease phenotype.^22^ This suggests a potential link between MTM1 and DNM2 in the liver in this animal model. However, cholestatic disease or other liver diseases have not been observed in mouse models of XLMTM or ADCNM. It would thus be useful to identify appropriate pre-clinical models to study liver disease in centronuclear myopathies. In skeletal muscle, both XLMTM and ADCNM share several pathological features including centrally or internally placed nuclei, myofiber smallness and mislocalization of organelles including mitochondria. This suggested a relationship between the pathogenesis of these two disorders, which revealed that myotubularin-deficient skeletal muscle contains high levels of dynamin 2 protein. As ADCNM is caused by increased activity of mutant dynamin 2 protein, treatments that decrease dynamin 2 have the potential to improve muscle disorders related to *DNM2* and *MTM1* mutations, and animal studies using DYN101-m (the mouse surrogate of the IMP DYN101) have been very successful in preventing or reverting disease phenotypes in both XLMTM and ADCNM models.^27–29^ Further study of the biological role myotubularin and dynamin 2 play in the liver will help identify whether XLMTM and ADCNM patients are at risk of similar types of liver dysfunction in these two diseases, and whether special monitoring or treatment measures should be considered in relation to liver function in one or both of these disorders.

This study has several strengths and limitations. First of all, the individuals screened were male and female adult patients, thus reducing a selection bias based on gender, and providing rare insight into adult XLMTM and ADCNM patients. Furthermore, several study subjects had one or more unscheduled visits providing more measurements of liver function. Unfortunately, several data points were missing from one study site, and primary liver imaging data were not collected centrally. Liver imaging by ultrasound and elastography, as well as bilirubin, bile acid and LDH measurements were added as an amendment during the trial and therefore not all subjects underwent these assessments. However, albeit incomplete, these additional measurements provide an important dataset shared here to support future therapeutic developments in this field. At the same time, the amendments to the protocol can be considered a strength, ensuring that the patients were safe throughout the trial.

We recommend assessment of liver function and structure at baseline in all individuals, and to lower the maximal amount of alcohol consumption, following the WHO recommendations^30^: The MTM-CNM Liver Collaborative Working Group has composed an expert-opinion fact sheet for patients: ‘Liver Considerations for Myotubular & Centronuclear Myopathy’.^31^

To conclude, the observations in Unite-CNM have provided an overview of hepatic involvement in a large, unselected group of CNM patients with two different genetic causes. Findings from the Unite CNM and gene therapy AT132 clinical studies in CNM and/or XLMTM patients suggest an underlying liver pathology may impact tolerability of therapeutic approaches, and will be important to consider for future trial design and clinical management. The results of DNM2 protein expression warrant further investigations on the role of DNM2 in the liver if it is to be used as a therapeutic target.

## Supplemental Material

sj-docx-1-jnd-10.1177_22143602251329215 - Supplemental material for Liver function in X-linked myotubular myopathy and autosomal dominant centronuclear myopathy: Data of the unite-CNM study Supplemental material, sj-docx-1-jnd-10.1177_22143602251329215 for Liver function in X-linked myotubular myopathy and autosomal dominant centronuclear myopathy: Data of the unite-CNM study by S Colombo, BS Cowling, L Eyler, D Nijkamp, C Freitag, L Thielemans, K Bouman, J Baets, J Vissing, R Quinlivan, M Guglieri, F Montagnese, U Schara-Schmidt, A Dhawan, MW Lawlor and NC Voermans in Journal of Neuromuscular Diseases
