# Supplementary material for: Liver function in X-linked myotubular myopathy and autosomal dominant centronuclear myopathy: Data of the unite-CNM study
Source: J Neuromuscul Dis. 2025 Apr 24;12(4):497–512. doi: 10.1177/22143602251329215 (PMC13142885; doi:10.1177/22143602251329215)
Supplement: sj-docx-1-jnd-10.1177_22143602251329215 - Supplemental material for Liver function in X-linked myotubular myopathy and autosomal dominant centronuclear myopathy: Data of the unite-CNM study [file sj-docx-1-jnd-10.1177_22143602251329215.docx]

**Supplemental data: Methods**

Western Blot on human liver

Fifteen human autopsy liver tissues (6 healthy pediatric livers, 5 healthy adult livers, and 4 XLMTM pediatric livers) were available for protein analysis. The samples were lysed in Tissue Protein Extraction Reagent (T-PER) (#78510 – Thermo Scientific) buffer supplemented with Mix Halt Protease Inhibitor Cocktail (1/100) and Mix Halt Phosphatase Inhibitor Cocktail (1/100) during 1 hour at 4°C under agitation. Samples were centrifuged at 10,000 rpm for 5 minutes at 4°C and supernatants were collected. Protein concentration was determined with the BioRad DC protein Assay kit (#500-0113– BioRad) and the Multiskan Go machine with the SkanIT RE software. Samples were diluted at 1 ng/µL in Total T-PER buffer, NuPAGE LDS sample buffer (#NP0007 – Invitrogen) and DTT 0.5M and denatured at 70°C for 10 minutes. 12.5 ng of protein were loaded, as well as a DNM2 recombinant protein standard curve (0.5 ng, 0.25 ng, 0.1 ng, 0.06 ng, 0.03 ng, 0 ng) in 4-15% precast polyacrylamide gels and separated by electrophoresis (Bio-Rad) during 1 hour at 100V and transferred on nitrocellulose membrane for 7 min (Trans-Blot Turbo Transfer System, #1704150 – BioRad). After the transfer, total proteins were stained with Ponceau (#P7170-1L – Sigma) for 2 minutes and the membranes were washed with water for 2 minutes. Membranes were blocked for 5 minutes at room temperature (RT) in EveryBlot Blocking Buffer (#12010020 – Biorad) before an incubation for 1 hour at RT with primary mouse monoclonal antibodies against DNM2 (1/1000, #PA5-19800 – Thermo Scientific), or GAPDH (1/1000, #MA515738 – Invitrogen) diluted in blocking buffer. After three washes during 5 minutes in TBST (TBS: #1706435 – Biorad + Tween 20: #BP337-500 – Fisher Bioreagents), membranes were incubated with goat anti-mouse secondary antibody coupled to horseradish peroxidase (1/1000 for GAPDH, #32430 – Invitrogen) and goat anti-rabbit secondary antibody coupled to horseradish peroxidase (1/1000 for DNM2, #32460 – Invitrogen) for 1 hour at RT. Nitrocellulose membranes were then imaged with a Fusion FX imager (Vilber) after an incubation with ECL Immobilon (#WBKLS0100 – Millipore) during 5 minutes for DNM2 membrane and ECL Pierce (#32106 – Thermo Scientific) during 3 minutes for GAPDH membrane. GAPDH was used as a loading control and the protein quantification was done using the DNM2 standard curve.

Animals

*Mtm1* knockout mice (*Mtm1* KO) or wild-type (WT) 129SvPAS mice were generated by crossing *Mtm1* heterozygous females with WT males. Mice were handled according to the French and European legislation on animal care and experimentation. Protocol was approved by the institutional Ethics Committee (Protocol No.: N°5453-2016052510176016). Mice were kept on 12h day light and 12h cycle and given free access to standard food. All mice analyzed were male. WT mice at 2 weeks of age were untreated. All other mice were injected with one dose of DYN0-m, a control antisense oligonucleotides not targeting any gene, and known to have no effect in mice (used as a control), at 3 weeks old.[1]

RT-qPCR on mouse muscle and liver

Total RNA was isolated from gastrocnemius muscle (GAS) and liver using MagMAX™ mirVana™ Total RNA Isolation Kit (#A27828 - Applied Biosystems). RT-PCR was carried out on 500 ng RNA using SuperScript™ IV Reverse Transcriptase (#18090050 - Thermofischer Scientific). qPCR was performed in Quant Studio 3 Real-Time PCR System (ThermoFischer Scientific) using the following primers: *Tbp* Forward 100 μM 5’-ACCGTGAATCTTGGCTGTAAAC-3’; *Tbp* Reverse 100 μM 5’-GCAGCAAATCGCTTGGGATTA-3’; *Dnm2* Forward 100 μM 5’-ACCCCACACTTGCAGAAAAC-3’; *Rpl27* Forward 100 μM 5’-AAGCCGTCATCGTGAAGAACA-3’; *Rpl27* Reverse 100 μM 5’-CTTGATCTTGGATCGCTTGGC-3’; *Dnm2* Forward 100 μM 5’-ACCCCACACTTGCAGAAAAC-3’; *Dnm2* Reverse 100 μM 5’CGCTTCTCAAAGTCCACTCC-3’; mixed in PowerUp™ SYBR™ Green Master Mix (#A25918 - ThermoFisher Scientific).

**Reference**

1. Koch C, Buono S, Menuet A, Robé A, Djeddi S, Kretz C, et al. Myostatin: a Circulating Biomarker Correlating with Disease in Myotubular Myopathy Mice and Patients. Mol Ther Methods Clin Dev. 2020;17:1178-89.
